# Editorials

**DOI:** 10.1155/S1463924684000018

**Published:** 1984

**Authors:** Peter B. Stockwell


Journal of Automatic Chemistry, Volume 6, Number 1 (January–March 1984), pages 1–2

### Editorials

### 1984—and all that?

Despite George Orwell's predictions we have arrived into 1984 relatively safely. What does the year promise? Will robotics take over or will they be slowly integrated into our lifestyle? Whilst it is obviously exciting to look at new developments it is all too easy to forget the lessons of the past. One of our Editorial Advisory Board members, Dr F. L. Mitchell, retired in 1983, only, I predict, to enter a more hectic phase of his life. In this issue he shares with us his reflections of 33 years' involvement in laboratory automation.

Some time ago I was involved as external examiner for an MSc thesis on the history of instrumentation, particularly of automation. This seemed to be worthy of a wider readership and the article by R. Stanley in this number is the first paper we have published on the topic.

From these two reflections on the past I hope that we will be better equipped to employ the new technologies to their maximum advantages

**Peter B. Stockwell**
*Editor*

---

### Reflections on the laboratory automation scene 1950–1983

It is difficult to envisage a time in the future when change in the laboratory will have an impact approaching that experienced over the last 33 years. The somewhat primitive approaches to mechanization [1] introduced in the late 1950s have been replaced in recent years by the dramatic application of microcomputers; over the whole period, however, milestones in the development of analytical prowess have been passed at a rate never previously experienced. We tend to forget that many household terms now familiar to all analysts were unheard of in the 1950s, at least with any remote possibility of connection with chemical analysis, be it for routine or research purposes. The list is impressively long and includes chromatography in its various forms (liquid—high and low pressure, paper, thin-layer and gas), electrophoresis in its many forms, immunoassay also in its various forms, mass spectrometry, nuclear magnetic resonance, and what has been called 'solid state chemistry'. Currently, exciting developments may be expected from the intensive work now being concentrated on the various systems applicable in the so-called 'probe approach to analysis', for example ion-selective electrodes and field-effect transistors.

Though these new principles for analysis cannot in themselves be considered as automation [2] as defined in the proposals by IUPAC, they do come under the heading of mechanization. Since the proposed definition of automation is somewhat restrictive, perhaps in the present context we should be really considering mechanization and if we do this the principles previously mentioned have certainly profoundly affected the path which automation, mechanization, or whatever, has taken and will take.

In the 1950s and 1960s we were concerned to replace the human drudgery involved in carrying out the many manipulations required for classical quantitative analysis, and the continuous-flow, flow-injection and discrete approaches to automation have served well in this regard. The early machines had to operate without computer assistance but now it is generally accepted that two microprocessors are required, one to operate the machine itself and the other to deal with specimen identification, data handling, quality control etc.

Some might say that in many circumstances the application of microcomputers has been overdone, for example it is now difficult to obtain a spectrometer without elaborate computer-controlled functions, mostly developed by manufacturers desperate to maintain their share of a market which demands laboratory equipment to be ever-more complex. It has been said that one manufacturer felt it necessary to advertise his relatively simple centrifuge as being based on a computer—a microprocessor was built into the base but it was not connected to the mechanism. Do we need all the gadgets now found on spectrometers for many spectrophotometric measurements? Perhaps the answer is that we do not, but neither do we really need many of the gadgets which come with the modern car. How many of those concerned with work involving HPLC, atomic absorption or any form of data processing, would lightly give up their instrument-controlling microprocessors or number-crunching computers?

The value of automation can be categorized under two headings—the first and most obvious includes the elimination of human drudgery, cost reduction and improved speed for procedures previously done by hand; the second, and possibly the more important, covers the improvement of quality and the possibility of doing things which would otherwise be impossible or impractical. The improvement in quality stemmed at first from the elimination of human error in sample identification, the addition of reagents, reading and calculation of results etc., but later came the automatic applications of such items as regular automatic calibration, drift correction and malfunction reporting. There is an ever-increasing number of functions which are now accepted as commonplace but which were inconceivable before the advent of the principle of mechanization and the means by which it is achieved. The Vidicon tube allows the collection of an incredible amount of data in the short time available during fast reactions; much mass spectrometry work would be impractical without the rapid data collection, processing and handling made possible by computer; much NMR work can only be done because data can be collected and averaged over hours or even days. Finally, to come to the practicalities of everyday life, thanks to solid phase layer chemistry, the doctor on his rounds will soon be able to make the most complex measurements at the bedside using equipment which he can carry in his pocket or at least in the glove-box of his car. These developments, together with the almost limitless potential which might be foreseen for the application of probe analysers, promise to change the function of the chemical analyst just as surely as the advent of home television and the domestic washing-machine has led to the demise of the cinema and the commercial laundry.

Nowhere is the future scenario so clear as in clinical chemistry where a very short time ago the efficiency of the large multichannel analyser seemed to pave the way long into the future for a trend towards centralization, with laboratories working on factory principles. This trend has in fact never been reversed since physicians at the end of the last century began to find that pathology increasingly required special skills and instruments which they themselves could not or did not possess. Now, in chemical pathology (or clinical chemistry), which has overtaken in size all other branches of pathology, automatic chemical analysers are becoming so small, reliable and simple to operate that the physician (or his secretary) can own and operate them, thereby eliminating that expensive and time-consuming loop of activity which is involved in sending a specimen to a distant laboratory and receiving a result. The analogy with the family laundry is frighteningly clear. Those analysts now in clinical chemistry could well watch the clinical chemists carefully since they started laboratory automation and it will be in their profession that any resulting changes in professional organization first become apparent.

In these matters we are dealing with the advance (?) of civilization and the consequences are inexorable, just as surely as were those which followed the industrial revolution or those which we are now experiencing as a result of factory and office automation. Old industries and ways of life disappear only to be replaced by new; for the analyst some time ago many of the exciting developments of his trade moved from his immediate environment to that of the commercial producers of analytical kits and equipment, whose laboratories are almost the only ones where the considerable finance is available to execute the necessary developments. Perhaps it is to openings in places such as these that analysts should be looking for an exciting future—just as the future for many laundry workers now lies in factories making home washing-machines and their supporting industries.

The effects of mechanization on chemical analysis over the last 33 years have been dramatic and exciting for all concerned, and improvements in efficiency are widely recognizable in all aspects of chemistry and biochemistry, with new areas of application opening up at an ever-increasing rate. Many breakthroughs in the understanding of important mechanisms have followed in the wake of new developments in analysis and many would not have been possible without such breakthroughs. A quick survey of the last 100 articles in *Clinical Science*: 76, used measurements of endogenous compounds; seven, measurements of respiratory gases; 26, measurements of drugs; and 47, physical measurements (volume, pH, flow rate etc.). A measurement of some sort was the basis of all.

A senior medical research director recently said that all the necessary development in analysis required for medical research had taken place and no further investment was necessary. Only time will show whether that is true or not.

### References

1. Proposed IUPAC definition of mechanization—'The use of devices to replace, refine, extend or supplement human effort'.
2. Proposed IUPAC definition of automation—'The process whereby human intervention is replaced by devices which are regulated by feedback so that they are self-monitoring or self-adjusting'.

**F. L. Mitchell**
*Hertfordshire, UK*

### CONFERENCE ANNOUNCEMENT

*'Chromatography and Mass Spectrometry'*

The Italian Group for Mass Spectrometry in Biochemistry and Medicine is organizing the 2nd International Conference on Chromatography and Mass Spectrometry in Biomedical Sciences for 10–20 June 1984 in Milan, Italy. The conference will illustrate and discuss the latest aspects of chromatography, mass spectrometry and chromatography mass spectrometry and their areas of application, including biochemistry, medicine, toxicology, drug research, nutrition science and food safety, forensic science, clinical chemistry and pollution. A major aim is to stimulate the exchange of information among scientists working in different fields. As well as lectures by invited speakers, contributed papers and discussions, there will be facilities available for participants to display poster communications. Also planned are a book exhibition and displays of manufacturers' literature on chromatography and mass spectrometry.

*For further details contact either Dr Alberto Frigerio, President: Italian Group for Mass Spectrometry in Biochemistry and Medicine, Via Eustachi 36, I 20129 Milan, Italy; or Dr Hubert Milon, P O Box 88, CH 1814 La Tour-de-Peilz, Switzerland.*

### READER ENQUIRY SERVICE

Each advertisement and each editorial item in the Product News section in *Journal of Automatic Chemistry* carries a number which corresponds with a number on the Reader Enquiry Card bound into every issue.

If there are any items on which you would like further information, circle the relevant number on the card, indicate the volume and issue number, fill in your name and address, and return the card to Taylor & Francis Ltd. Your card will then be forwarded to the company/ies concerned for action.

*Through using the Reader Enquiry Service, you can obtain information on a variety of products through one simple operation.*

